# Knockdown of long non-coding RNA HOTAIR increases miR-454-3p by targeting Stat3 and Atg12 to inhibit chondrosarcoma growth

**DOI:** 10.1038/cddis.2017.31

**Published:** 2017-02-09

**Authors:** Xing Bao, Tingting Ren, Yi Huang, Kunkun Sun, Shidong Wang, Kuisheng Liu, Bingxin Zheng, Wei Guo

**Affiliations:** 1 Musculoskeletal Tumor Center, Peking University People's Hospital, Beijing, People's Republic of China; 2 Beijing Key Laboratory of Musculoskeletal Tumor, Beijing, People's Republic of China; 3 Department of Pathology, Peking University People's Hospital, Beijing, People's Republic of China

## Abstract

Current practices for the therapy of chondrosarcoma, including wide-margin surgical resection and chemotherapy, are less than satisfactory. Recently, emerging evidence has demonstrated that long non-coding RNAs (lncRNAs) have an essential role in the initiation and progression of tumors. As a typical lncRNA, HOTAIR is significantly overexpressed in various tumors. However, the function and potential biological mechanisms of HOTAIR in human chondrosarcoma remain unknown. Quantitative RT-PCR demonstrated that HOTAIR expression was upregulated in chondrosarcoma tissues and cell lines. High HOTAIR expression is correlated with tumor stage and poor prognosis. Functional experiments reveal that HOTAIR knockdown leads to growth inhibition of human chondrosarcoma cells *in vitro* and *in vivo*. In addition to cycle arrest and apoptosis, knockdown of HOTAIR inhibits autophagy, which favors cell death. Mechanistically, we demonstrated that HOTAIR induced DNA methylation of miR-454-3p by recruiting EZH2 and DNMT1 to the miR-454-3p promoter regions, which markedly silences miR-454-3p expression. Further analysis revealed that STAT3 and ATG12 are targets of miR-454-3p, initiate HOTAIR deficiency-induced apoptosis and reduce autophagy. Collectively, our data reveal the roles and functional mechanisms of HOTAIR in human chondrosarcoma and suggest that HOTAIR may act as a prognostic biomarker and potential therapeutic target for chondrosarcoma.

Chondrosarcoma is the second most commonly occurring primary bone tumor.^1, 2, 3^ Currently, chondrosarcoma remains largely incurable because of poor prognosis and a high rate of recurrence.^4^ Thus, the development of innovative targeted molecular therapies for this disease is imperative. Long non-coding RNAs (lncRNAs) have been demonstrated to have pivotal roles in various human diseases, including tumors.^5, 6^ However, which lncRNAs are involved in human chondrosarcoma initiation and progression remains unknown.

HOTAIR is a well-studied lncRNA that is located on human chromosome 12q13 within the anti-sense strand of the HOXC gene cluster and has been shown to alter gene expression through the recruitment of chromatin modifiers.^7^ The important epigenetic modification modes of DNA methylation and histone methylation are known to directly regulate the expression of various tumor-related genes.^8, 9^ Aberrant DNA methylation and histone methylation, mediated by HOTAIR, have been found in many types of tumors.^10, 11^ The high expression of HOTAIR in tumors is indicative of a poor prognosis.^12, 13^ Moreover, HOTAIR knockdown induced apoptosis in many tumor cell lines, and ectopic expression of HOTAIR reduced this effect.^14, 15, 16, 17^ However, few studies have examined why HOTAIR knockdown results in tumor cell apoptosis, and whether HOTAIR modulates autophagy in human chondrosarcoma cells remains unknown.

Recent studies have shown that HOTAIR has a critical role in human tumors by inhibiting microRNAs (miRNAs).^17, 18, 19^ MiRNAs are small non-coding RNAs 20–25 nucleotides in length that regulate gene expression at the post-transcriptional and/or transcriptional level, mainly by targeting the 3'-untranslated regions of mRNAs.^20, 21^ Abnormal expression of miRNAs has been found in various types of tumors.^22, 23, 24, 25^

It has been reported that several mechanisms lead to miRNA dysregulation, including aberrant histone acetylation, histone methylation and DNA methylation of CpG islands.^26, 27^ However, whether HOTAIR regulates miRNA expression and function, and the underlying molecular mechanisms remain largely unknown in human chondrosarcoma.

In this study, we found that the level of miRNA miR-454-3p increased after HOTAIR knockdown, and its upregulation repressed the translation of its target, the transcription factor signal transducer and activator of transcription 3 (Stat3), which has an important role in tumor proliferation, and another target, autophagy-related gene 12 (ATG12), which is an autophagy marker that is significant for the resistance to apoptosis in many types of tumors.

## Results

### HOTAIR expression in chondrosarcoma and its relationship to patient survival

**Figure 1:**
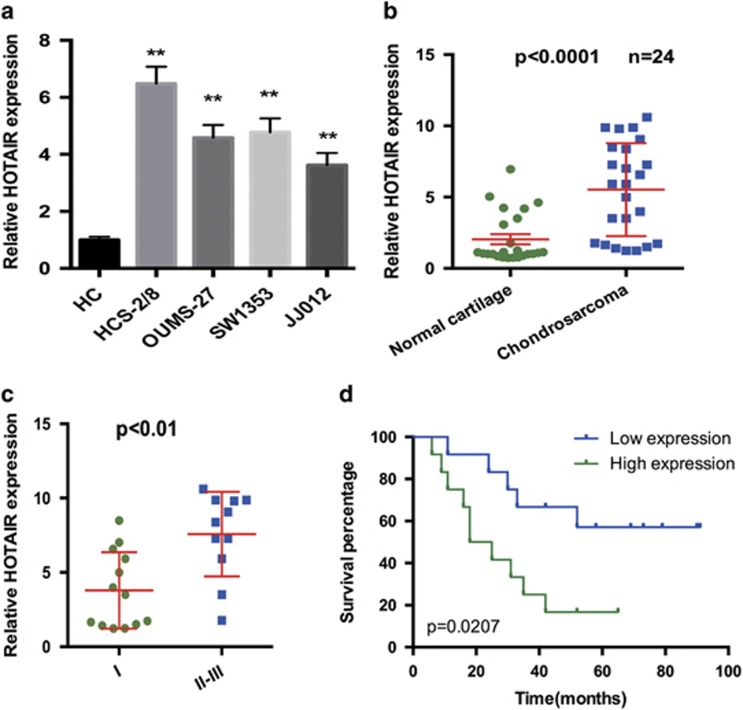
HOTAIR expression in chondrosarcoma and its relation to patient survival. (**a** and **b**) HOTAIR expression was upregulated in both chondrosarcoma tissues and cell lines. (**c**) HOTAIR expression was higher in high-grade (grades II+III) chondrosarcoma tissues compared with low-grade (grade I) chondrosarcoma tissues. The median expression level was used as a cutoff. (**d**) Overall survival of 24 chondrosarcoma patients. Data represent the mean±S.D. (*n*=3) in **a**. **P<0.01 by Student's *t*-test. The bars illustrated S.E.M. and the significant differences between samples were analyzed using Student's *t*-test in **b** and **c**. All experiments were performed in three biological repeats

Previous studies have indicated that HOTAIR expression was upregulated in various tumors.^12, 13^ Our data validated that HOTAIR was significantly increased in chondrosarcoma cells (Figure 1a). We also found that HOTAIR expression was increased in chondrosarcoma tissues compared with normal cartilage tissues (Figure 1b). Moreover, HOTAIR expression was higher in high-grade (grades II+III) chondrosarcoma tissues compared with low-grade (grade I) chondrosarcoma tissues (Figure 1c). Next, we examined the correlation between HOTAIR expression and chondrosarcoma patient prognosis. Kaplan–Meier survival analysis showed that the overall survival time of patients with high HOTAIR expression was significantly shorter than that of patients with low HOATAIR expression (Figure 1d).

### HOTAIR knockdown directly leads to growth inhibition of human chondrosarcoma cells via G0/G1 arrest and apoptosis

**Figure 2:**
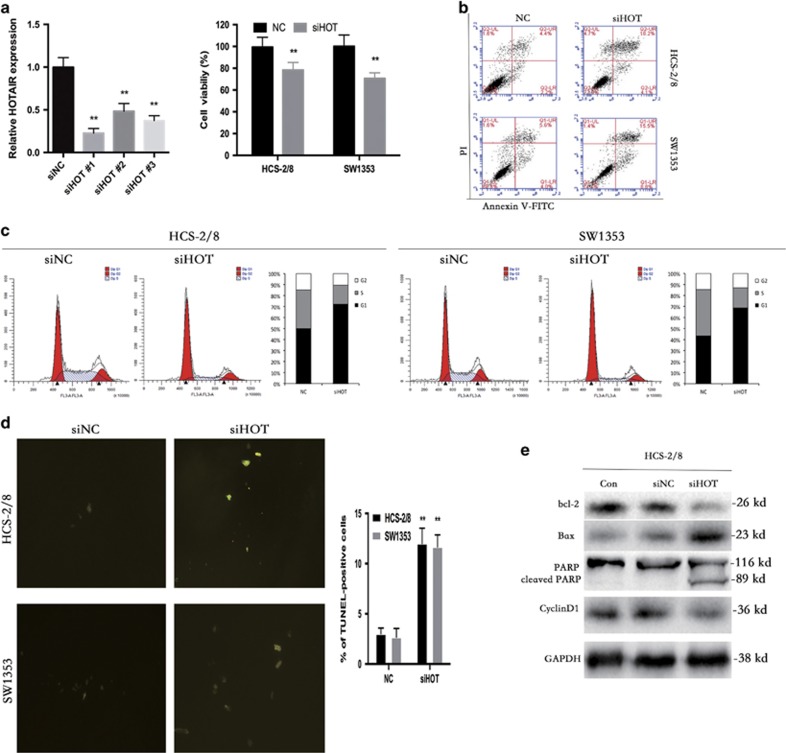
HOTAIR knockdown leads to direct growth inhibition of human chondrosarcoma cells through G0/G1 arrest and apoptosis. (**a**) Three HOTAIR siRNA sequences were used to downregulate HOTAIR in HCS-2/8 cells, the level of HOTAIR was significantly decreased when transfected with siHOTs (left). Cell viability was assessed by CKK-8 assay (right). The data represent the mean±S.D. of three independent experiments. **P<0.01 by Student's *t*-test. (**b**) The percentage of apoptosis was determined by FCM analysis. (**c**) Cell cycle analysis was performed using FCM. (**d**) TUNEL staining of HCS-2/8 cells at 48 h post-transfection with siHOT. Positive cells were labeled with TUNEL (green) (magnification × 400). Quantification of the number of TUNEL-positive cells (graph on the right), plotted as the percentage of TUNEL-positive cells. **P<0.01 by Student's *t*-test. (**e**) Effects of HOTAIR knockdown on G0/G1 phase-related and apoptosis-related proteins was assayed by western blot. Representative data from one of three independent experiments are shown in **b**–**e**

To determine whether HOTAIR is essential for chondrosarcoma cell survival and proliferation, we transfected three siRNA sequences targeting HOTAIR (siHOT) or scrambled siRNA (siNC) into HCS-2/8 and SW1353 cells. Decreased cell viability was observed by a CKK-8 assay (Figure 2a). Apoptosis assays were carried out at 48 h post-transfection. Substantial apoptosis was observed by flow cytometry (FCM) in these two cell lines (Figure 2b).

Our analysis of cell cycle assay revealed that chondrosarcoma cells were mostly arrested in the G0/G1 phase, implying that there was a reduction in the number of dividing tumor cells following the knockdown of HOTAIR (Figure 2c).

Terminal deoxynucleotidyl transferase-mediated nick end labeling (TUNEL) staining was performed to confirm the induction of apoptosis (Figure 2d).

In addition, the ratio of Bax protein to both Bcl-2 and caspase-cleaved PARP – key executors of cell apoptosis – increased in chondrosarcoma cells transfected with siHOT, as analyzed by western blot. In contrast, the level of cyclin D1, a G0/G1 phase-related protein, decreased (Figure 2e).

Altogether, these data demonstrate that HOTAIR is vital for chondrosarcoma cell survival and downregulation of HOTAIR leads to tumor cell apoptosis.

### HOTAIR knockdown-induced inhibition of autophagy indirectly promotes chondrosarcoma cell apoptosis

**Figure 3:**
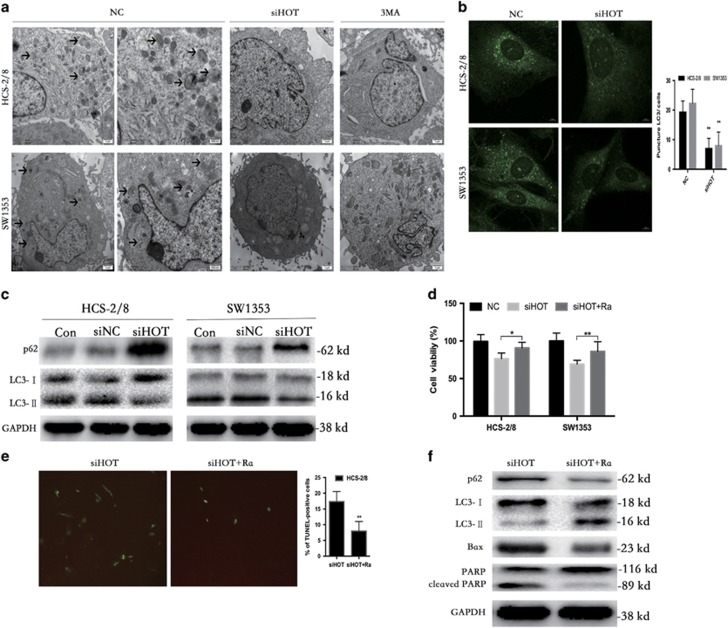
HOTAIR knockdown-induced inhibition of autophagy promotes chondrosarcoma apoptosis. (**a**) Representative TEM images depict the ultrastructures present during autophagy in HCS-2/8 and SW1353 cells transfected with HOTAIR siRNA or siNC for 24 h. The images show autophagic vacuoles (arrows) observed in control cells. No or few autophagic vacuoles were observed in siHOT and 3MA-treated cells. (**b**) Cells transfected with siHOT exhibited a punctate pattern of LC3-II fluorescence, with reduced LC3-II compared with autophagosomes. (**c**) Western blot analysis was used to evaluate the expression of LC3 and p62. (**d**) CCK-8 assay revealed that treatment of cells with rapamycin increased the number of viable HOTAIR knockdown cells. (**e**) TUNEL staining of HOTAIR knockdown cells was markedly decreased in the presence of rapamycin. (**f**) Treatment with rapamycin markedly increased LC3-II, and decreased Bax and cleaved PARP expression. Data are presented as mean±S.D. (*n*=3). *P<0.05, **P<0.01

Autophagy-associated cell death is another type of programmed cell death. To investigate whether HOTAIR is involved in autophagy, transmission electron microscopy (TEM) was performed to observe the ultrastructures present during autophagy. Chondrosarcoma cells transfected with siHOT and those treated with 3MA exhibited few autophagic vacuoles compared with typical autophagic vacuoles, and a distinct double membrane was present in the control cells (Figure 3a).

LC3 is a specific marker of autophagy initiation and is processed from LC3-I to LC3-II during autophagy. Therefore, the expression of LC3-II, as visualized by immunofluorescence, can be used to track changes in autophagosome formation in chondrosarcoma cells. As shown in Figure 3b, downregulation of HOTAIR induced LC3 accumulation in chondrosarcoma cells. Cells transfected with siHOT exhibited few punctate pattern of LC3-II fluorescence, showing a reduction of LC3-II in autophagosomes. Decreased LC3-II expression and an accompanying increase in p62 expression were clearly detected by western blot (Figure 3c).

**Figure 4:**
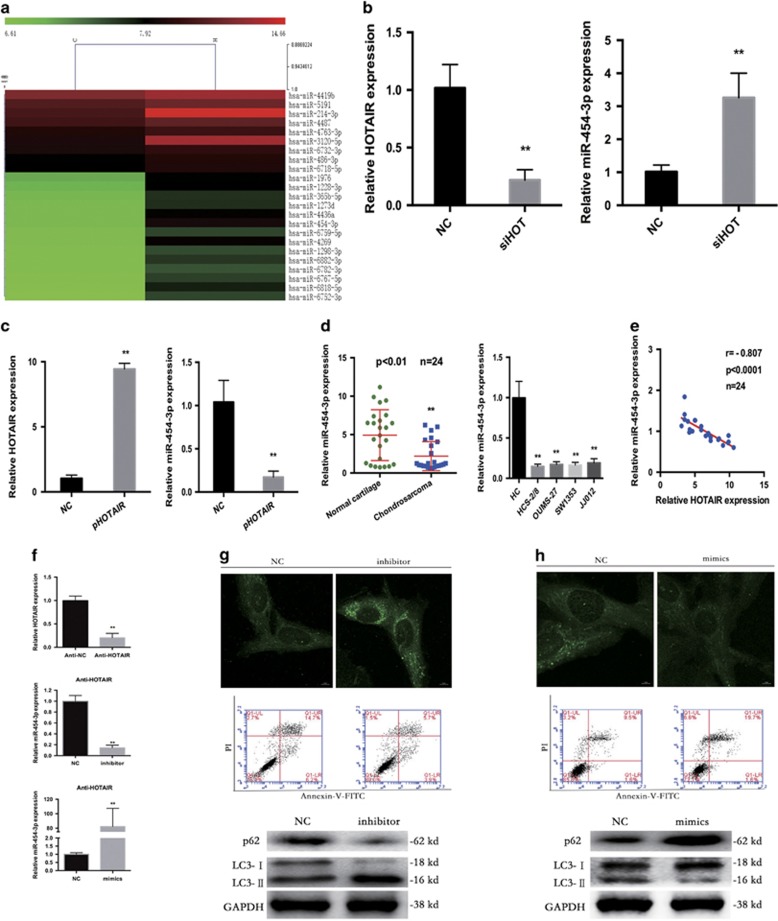
HOTAIR mediated chondrosarcoma cell growth by negatively regulating miR-454-3p expression in chondrosarcoma. (**a**) Heat map of genes exhibiting significant induction (red) at 24 h after siHOT transfection into HCS-2/8 cells (expressed as a ratio to HCS-2/8 cells transfected with siNC; fold change >2, *P*-value <0.05, data are log_2_ transformed). (**b**) qRT-PCR demonstrated that HOTAIR expression was downregulated by siHOT and that miR-454-3p expression was markedly enhanced. (**c**) MiR-454-3p was downregulated in chondrosarcoma cells with HOTAIR overexpression. (**d**) MiR-454-3p was significantly downregulated in chondrosarcoma tissues compared with normal cartilage tissues. The bars illustrated S.E.M. **P<0.01 by Student's *t*-test. (**e**) The correlation between HOTAIR and miR-454-3p expression levels in 24 chondrosarcoma tissues. (**f**) HOTAIR and miR-454-3p expression levels checked by qRT-PCR. (**g**) MiRNA inhibitors markedly increased autophagy and rescued apoptosis caused by HOTAIR knockdown. (**h**) MiRNA mimics reduced autophagy and increased apoptosis

Autophagy can either inhibit or promote tumor cell growth in different cellular contexts.^28, 29, 30, 31^ Given that manipulating autophagy may improve the efficacy of anticancer therapeutics,^32, 33^ we were eager to determine whether the HOTAIR knockdown-induced inhibition of autophagy in chondrosarcoma favored cell survival or cell death. We used the autophagy inducer rapamycin, an mTOR inhibitor that exerts an autophagy-inducing effect. Treatment of cells with rapamycin increased the number of viable HOTAIR knockdown cells as assayed by CCK-8 (Figure 3d). TUNEL staining of HOTAIR knockdown HCS-2/8 cells was markedly decreased in the presence of rapamycin (Figure 3e). Treatment with rapamycin obviously increased LC3-II and decreased Bax and cleaved PARP expression, indicating that autophagy inhibition induced by HOTAIR knockdown results in HCS-2/8 cell apoptosis (Figure 4f).

### HOTAIR mediated chondrosarcoma cell growth by negatively regulating miR-454-3p expression in chondrosarcoma

To determine why HOTAIR is crucial for tumor cell survival, we performed a miRNA microarray assay to screen for miRNAs regulated by HOTAIR. Total RNA from HCS-2/8 cells transfected with siHOT for 48 h was extracted and analyzed. We found that 23 miRNAs were upregulated, and among the changes in miRNA triggered by siHOT, a marked increase in miR-454-3p was observed, which was the only one downregulated both in chondrosarcoma tissues and cell lines (Figure 4a).^23^

To further verify the negative regulation of miR-454-3p by HOTAIR, we silenced HOTAIR using siHOT and found that HOTAIR expression was downregulated and that miR-454-3p expression was markedly enhanced in HCS-2/8 cells (Figure 4b). In addition, miR-454-3p was downregulated in HCS-2/8 cells with HOTAIR overexpression (Figure 4c).

Furthermore, miR-454-3p expression was detected by quantitative RT-PCR (qRT-PCR) in the same cohort containing 24 pairs of chondrosarcoma tissues and normal cartilage tissues, as shown in Figure 1b. These results showed that miR-454-3p were significantly downregulated in chondrosarcoma tissues compared with normal cartilage tissues (Figure 4d). Next, we measured the correlation between HOTAIR and miR-454-3p expression. Statistically, significant inverse interrelations were observed between HOTAIR and miR-454-3p expression in chondrosarcoma tissues (Figure 4e). These data reveal that HOTAIR negatively regulates miR-454-3p expression in chondrosarcoma cells.

Furthermore, miRNA mimics and inhibitors were used to explore the function of miR-454-3p. We transfected miRNA inhibitors and mimics into stable anti-HOTAIR HCS-2/8 cells (Figure 4f). MiRNA inhibitors markedly increased autophagy and rescued the apoptosis caused by the HOTAIR knockdown (Figure 4g). We transfected miR-454-3p mimics into HCS-2/8 cells and observed an increase in apoptosis and a reduction of autophagy (Figure 4h). These results suggest that miR-454-3p has a significant role in the HOTAIR knockdown-induced inhibition of cell growth in human chondrosarcoma.

### HOTAIR induced DNA methylation of miR- 454-3p through EZH2

**Figure 5:**
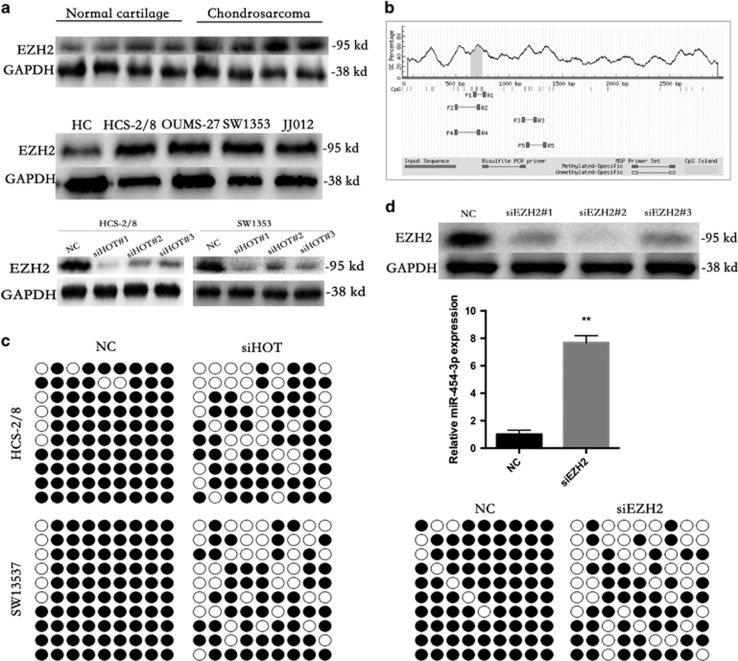
HOTAIR induced DNA methylation of miR-454-3p via EZH2. (**a**) EZH2 was upregulated in both chondrosarcoma cells and specimens(top and middle), and the level of EZH2 was sharply reduced in chondrosarcoma cells treated with siHOTs (bottom). (**b** and **c**) Downregulation of HOTAIR in HCS-2/8 and SW1353 cells significantly decreased DNA methylation levels at the CpG islands of miR-454-3p promoter regions. (**d**) Three EZH2 siRNA sequences were used to downregulate EZH2 in HCS-2/8 cells (top). The expression of miR-454-3p was improved by siEZH2 in HCS-2/8 cells (middle). SiEZH2 decreased the DNA methylation status of miR-454-3p promoter similar to siHOT (bottom). **P<0.01. Data represent the results of three independent experiments

Many studies have shown that HOTAIR is physically associated with EZH2.^34, 35, 36^ In our study, EZH2 was upregulated in both chondrosarcoma cells and specimens, and knockdown of HOTAIR led to repression of EZH2 (Figure 5a). EZH2 has been reported to bind and recruit DNMT1 to miRNA promoters.^27, 34^ As DNMT1 is known to mediate DNA methylation at the CpG islands of its target genes, we analyzed DNA methylation levels at the CpG islands of miR-454-3p promoter regions using bisulfate sequencing. The results revealed that the downregulation of HOTAIR in HCS-2/8 and SW1353 cells significantly decreased DNA methylation levels at the CpG islands of miR-454-3p promoter regions (Figures 5b and c).

To validate the direct regulatory role of EZH2 on miR-454-3p expression, quantitative real-time PCR was performed in chondrosarcoma cells. Our results revealed that the expression of miR-454-3p was increased in the presence of siEZH2 in HCS-2/8 cells, and siEZH2 decreased the DNA methylation status of miR-454-3p promoter similarly to siHOT (Figure 5d), indicating that miR-454-3p transcription may be epigenetically regulated by HOTAIR and its co-factor EZH2.

### Stat3 and Atg12 are targets of miR-454-3p and initiators of HOTAIR deficiency-induced apoptosis and autophagy reduction

**Figure 6:**
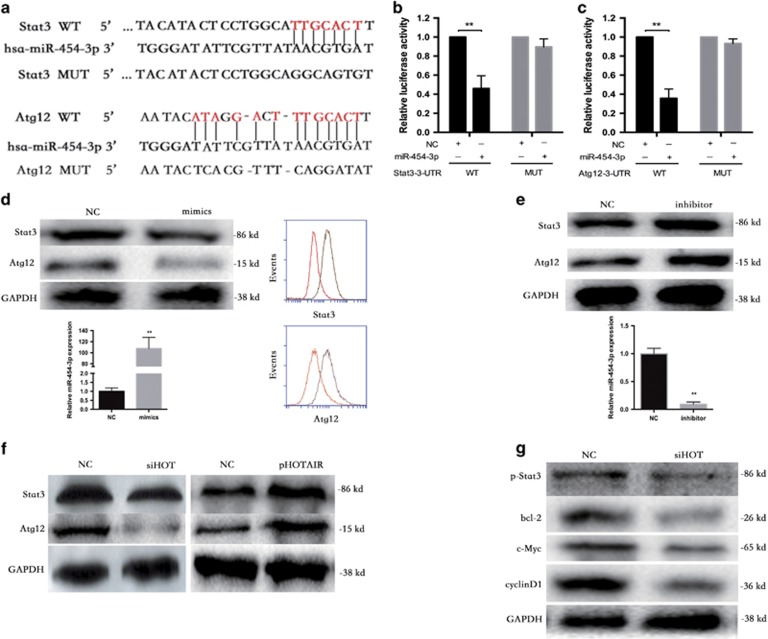
Stat3 and Atg12 are targets of miR-454-3p and initiators of HOTAIR deficiency-induced apoptosis and autophagy reduction. (**a**) The potential binding sites in the 3′-UTR of Stat3 and Atg12. (**b** and **c**) Dual-luciferase assays were performed in HCS-2/8 cells after co-transfection with wild-type or mutant Stat3 and Atg12 3′-UTR plasmids and with NC or miR-454-3p mimics. (**d**) Western blot and FCM demonstrated the significant downregulation of Stat3 and Atg12 in miR-454-3p-overexpressing cells (red) compared with NC cells (brown). (**e**) Upregulation of Stat3 and Atg12 in miR-454-3p knockdown cells. (**f**) Stat3 and Atg12 were suppressed by siHOTAIR and were promoted by pHOTAIR. (**g**) The expression levels of p-Stat3, bcl-2, c-Myc and cyclin D1 were downregulated by siHOT, suggesting the inactivation of Stat3 signaling. **P<0.01. Data represent the results of three independent experiments

As miRNAs function by targeting mRNAs, bioinformatics analyses using the target prediction tools miRNA, TargetScan and PicTar were used to identify potential binding sites in the 3′-UTRs of Stat3 and Atg12 (Figure 6a). We detected direct binding of miR-454-3p to the 3′-UTRs of Stat3 and Atg12 using a dual-luciferase reporter assay (Figures 6b and c) and observed significant downregulation of Stat3 and Atg12 in miR-454-3p-overexpressing HCS-2/8 cells via western blot assay and FCM (Figure 6d). We also found that miR-454-3p knockdown upregulated Stat3 and Atg12 expression (Figure 6e). In addition, Stat3 and Atg12 were suppressed by siHOTAIR and promoted by pHOTAIR at protein level (Figure 6f).

Our results reveal that Stat3 expression is repressed by siHOTAIR. We, therefore, wondered whether Stat3 signaling is involved in this process. As expected, the expression of p-Stat3, bcl-2, c-Myc and cyclin D1 was downregulated by siHOTAIR (Figure 6g), suggesting the inactivation of Stat3 signaling.

These data were in line with the results of FCM analysis, TUNEL assay and autophagy assay.

### HOTAIR knockdown results in growth inhibition of human chondrosarcoma cells via miR-454-3p upregulation and Stat3 signaling inactivation *in vivo*

**Figure 7:**
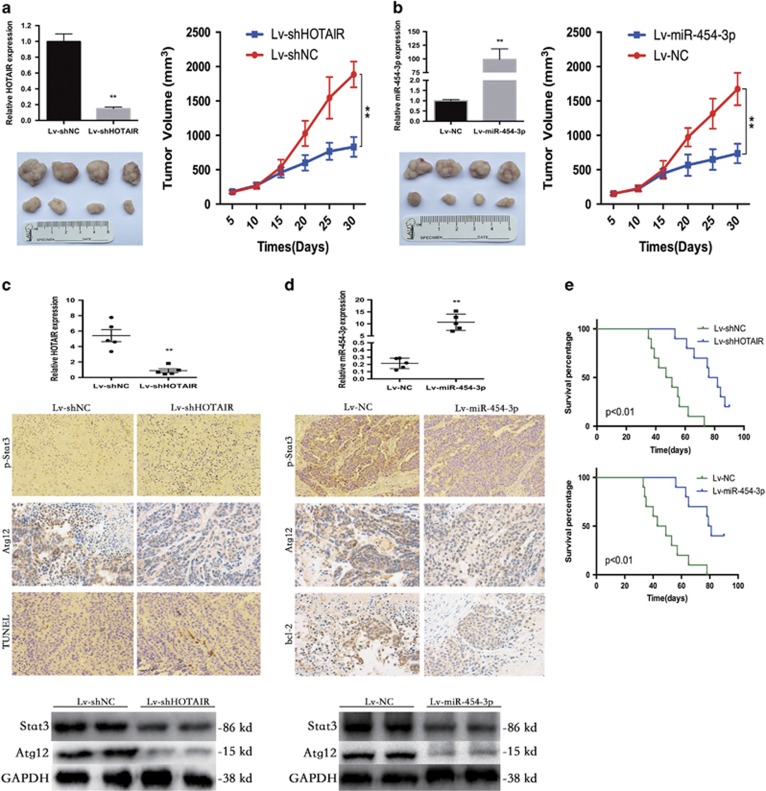
HOTAIR knockdown results in growth inhibition of human chondrosarcoma cells through miR-454-3p upregulation and Stat3 signaling inactivation *in vivo*. (**a**) The stable shHOTAIR group displayed a significant reduction in tumor volume and size. (**b**) A similar antitumor effect of miR-454-3p on chondrosarcoma cells was observed. Data are shown as mean±S.D. **P<0.01. (**c** and **d**) Xenograft tumors were removed and evaluated by immunohistochemistry, TUNEL and WB assay. (**e**) The stable expression of shHOTAIR and miR-454-3p in mice resulted in a significantly longer survival time compared with control mice. The experiment was repeated three times. **P<0.01

To further verify these *in vitro* findings, we used an *in vivo* xenograft model. HCS-2/8 cells stably infected with Lv-shHOTAIR or Lv-miR-454-3p were injected subcutaneously into the right armpit of nude BALB/c mice. Compared with the shNC group, the shHOTAIR group displayed a significant reduction in tumor volume and size (Figure 7a). A similar antitumor effect of miR-454-3p on chondrosarcoma cells *in vivo* is displayed in Figure 7b. The xenograft tumors were removed and evaluated by qRT-PCR, immunohistochemistry, TUNEL and WB (Figures 7c and d). Furthermore, the stable expression of shHOTAIR and miR-454-3p in the mice resulted in a significantly longer survival time compared with control mice (Figure 7e).

## Discussion

LncRNAs have recently been identified as novel regulators of transcriptional and epigenetic networks.^27^ HOTAIR, a classic trans-acting lncRNA, was found to be abnormally upregulated in several tumors, including hepatocellular carcinoma, colorectal cancers, pancreatic cancers, breast cancers, bladder cancers, cervical cancers and osteosarcoma.^7, 37, 38, 39, 40, 41, 42^ Many studies have revealed that HOTAIR acts as a potential antitumor target.

It has been confirmed that HOTAIR binds the histone modification complex PRC2 and LSD1 to regulate the expression of select genes and promote tumor cell migration and invasion.^12, 43, 44^ However, the functions of lncRNA in human chondrosarcoma remain largely unknown.

Our data verified that HOTAIR was overexpressed in chondrosarcoma specimens and cell lines. For the first time, we demonstrated that HOTAIR knockdown induces apoptosis and cell cycle arrest. Moreover, downregulation of HOTAIR inhibited autophagy, thereby promoting apoptosis in chondrosarcoma. It is well known that autophagy regulates apoptosis.^45, 46^ Previous studies have demonstrated that autophagy is common in some malignant tumors and the repression of autophagy sensitized apoptosis-resistant tumor cells to chemotherapy in several human tumors.^33, 47, 48^ Currently, data describing the effects of HOTAIR on autophagy are scarce. This study argues that siHOTAIR-mediated inhibition of autophagy is a cell death mechanism that promotes apoptosis, rather than a pro-survival mechanism.

To elucidate the molecular mechanism underlying HOTAIR function, a miRNA microarray assay and bisulfite sequencing analysis (BSP) were performed. In this study, we found that expression of miR-454-3p increased after HOTAIR knockdown, and we found that HOTAIR recruited EZH2 and DNMT1 to the promoter of miR-454-3p, increased DNA methylation at the miR-454-3p promoter regions and repressed miR-454-3p expression in chondrosarcoma.

The effects of HOTAIR on miR-454-3p are rooted in the association between HOTAIR and EZH2 and are abated by the inhibition of EZH2. Its overexpression has been observed in various tumors and is associated with poor prognosis.^34, 35, 36^ EZH2 has been reported to bind and recruit DNMT1 to the promoters of miRNAs, and DNMT1 is known to cause DNA methylation at the CpG islands of its target genes.^34^ In this study, we further validated that HOTAIR causes the anomalous expression of EZH2 and changes the DNA methylation level of miR-454-3p promoter regions.

MiR-454-3p has been reported to be dysregulated and to function as oncogene or anti-oncogene in various tumors.^49, 50, 51^ However, little is known about the function and underlying mechanism of miR-454-3p in chondrosarcoma.

According to our data, miR-454-3p is downregulated in chondrosarcoma tissues compared with normal cartilage tissues and is inversely correlated with HOTAIR expression in chondrosarcoma tissues. These data support the modulation of miR-454-3p by HOTAIR. The inverse effects of HOTAIR and miR-454-3p on cell growth further verified this negative regulation.

For the first time, we report that miR-454-3p functions as an anti-oncogene in chondrosarcoma cells by targeting Stat3 and Atg12. As a point of convergence for numerous oncogenic signaling pathways, Stat3 participates in cell growth through the regulation of cell proliferation and apoptosis via direct targets such as Bcl-2, c-Myc and cyclin D1.^52, 53^ ATG12 is a key factor involved in autophagosome formation.^54^ Recent studies have revealed that ATG12 is an important factor involved in radioresistance and chemoresistance.^46^ Our present data suggest that Stat3 signaling and Atg12 are downregulated by HOTAIR knockdown and ectopic miR-454-3p expression.

Taken together, this study reveals for the first time that miR-454-3p increases after HOTAIR knockdown to inhibit chondrosarcoma cell growth by targeting Stat3 and Atg12.

## Materials and methods

### Clinical specimens

Twenty-four chondrosarcoma tissues and adjacent normal cartilage tissues (located >3 cm away from the tumor) were collected under the protocols approved by the ethics committee of Peking University People's Hospital. None of the patients received antitumor treatment before surgery. Informed consents (written in the light of the ethical guidelines) were obtained from all the patients. The clinical characteristics of these patients were shown in Table 1. Fresh tissues were stored in liquid nitrogen before RNA extraction. Clinical and histopathologic information was recorded through a retrospective review of patient records.

### Cell culture and reagents

The human articular chondrocyte cell line HC-a (Sciencell, Carlsbad, CA, USA) was maintained in DMEM supplemented with 15% fetal bovine serum, plus antibiotics. SW1353 cells were obtained from American Type Culture Collection (ATCC, Manassas, VA, USA) and were maintained in L-15 medium (Gibco, Grand Island, NY, USA). OUMS-27 cells, HCS-2/8 cells and JJ012 cells were kindly gifted from Dr. J Block (Rush Medical College, Chicago, IL, USA) and were cultured in Dulbecco's modified Eagle's medium (Hyclone, Logan, UT, USA) supplemented with 10% fetal calf serum (Gibco) at 37 °C in a humidified atmosphere with 5% CO_2_.

Rapamycin and 3MA were purchased from Sigma Chemical Co. (St. Louis, MO, USA). The following antibodies were used in the experiments: anti-p-Stat3, anti-Stat3, anti-cyclin D1, anti-bcl-2, anti-Bax, anti-LC3, anti-p62 and anti-GAPDH were from Cell Signaling Technology (Beverly, MA, USA). Anti-c-Myc and anti-Atg12 was from Abcam (Cambridge, MA, USA).

### Transfection

SiEZH2, siHOTAIR and scrambled negative control siRNA (siNC) were synthesized in GenePharma (Suzhou, China). The sequences targeting HOTAIR and EZH2 are listed in Supplementary Table S1. The HOTAIR overexpression plasmid (pHOTAIR) was purchased from Addgene (Cambridge, MA, USA). MiRNA mimics and inhibitors were purchased from RiboBio (Guangzhou, China). RNAs were transfected into tumor cells using Lipofectamine3000 (Invitrogen, Carlsbad, CA, USA). MiR-454-3p and shHOTAIR stably expressed chondrosarcoma cells were infected with the lentivirus and selected with puromycin (1 *μ*g/ml) for 4 weeks.

### CCK-8 assay

Cells were plated in 96-well plates at a density of 5000 cells in 100 *μ*l medium per well 1 day before the experiment. The cell viability was examined by CCK-8 kit (Dojindo Laboratories, Kumamoto, Japan) according to the manufacturer's instruction.

### Western blot analysis

Equal amounts of proteins collected from different kinds of cell lysates were loaded on 10–15% SDS-PAGE gels using a NuPAGE system (Invitrogen) and then transferred onto PVDF membranes as previously described.^22^

### FCM experiments

Cells for cell cycle analysis were fixed in 70% ethanol, digested with RNase A and labeled with propidium iodide (PI). Apoptotic cells were analyzed with Annexin V/FITC kit (BD Biosciences, San Jose, CA, USA) according to the manufacturer's instructions and analyzed by FCM after compound treatment as previously described.^25^ Fluorescence-activated cell sorting was performed according to the manufacturer's instructions. Stat3 and Atg12-stained cells were quantified using FCM.

### Immunohistochemistry, immunofluorescence and TUNEL assay

IHC staining was performed as previously described.^27^ Paraffin sections were reacted with rabbit polyclonal anti-p-Stat3, anti-bcl-2 and anti-Atg12 antibodies (1:100 dilution). Sections stained with non-immune rabbit serum (1:200 dilution) in phosphate-buffered saline (PBS) instead of primary antibody served as negative controls. Cells exhibiting positive staining on cell membranes and in the cytoplasm and nucleus were counted in at least 10 representative fields (× 400 magnification) and the mean percentage of positive cells was calculated. Immunostaining was assessed by two independent pathologists blinded to clinical characteristics and outcomes.

For immunofluorescence assay of LC3, fixed cells were permeabilized with 0.1% Triton X-100 at room temperature for 15 min and incubated with anti-LC3 antibody overnight at 41C. Cells were washed three times with phosphate-buffered saline with Tween-20 and then incubated for 1 h with Cy3-conjugated goat anti-rabbit IgG at room temperature. The cells were then analyzed using confocal microscopy (FV10i, Olympus, Tokyo, Japan).

TUNEL assay was performed on cells. Apoptotic cells were detected using ApopTag plus peroxidase *in situ* apoptosis detection kit according to the manufacturer's instructions. Stained sections were visualized under fluorescence microscope.

### Transmission electron microscopy

After 48 h of HOTAIR siRNA treatment, TEM assay was performed on cells. For TEM assay, cells were digested with 0.25% trypsin and suspended at a concentration of 1.0 × 10^6^ per ml and fixation was carried out at 4 °C for 6 h with 1.5% glutaraldehyde. Later, ultrathin sections (100 nm) were prepared, stained with uranyl acetate and lead citrate and examined under an electron transmission microscope (H-600; Hitachi, Tokyo, Japan).

### Quantitative RT-PCR

The total RNA was extracted by Trizol reagent (Invitrogen). The reverse transcription was performed as described previously.^4, 15^ MiRNA qRT-PCR Primer Sets were purchased from RiboBio. Other genes' primer sequences are provided in Supplementary Table S2. U6 or GAPDH were used as endogenous controls.

### MiRNA microarray assay

Total RNA from cells transfected with siHOT for 24 h was extracted using RNeasy mini kit (Qiagen, Venlo, The Netherlands), and reverse transcribed according to the manufacturer's instructions (Fermentas, Waltham, MA, USA). The miRNA microarray analysis was carried out by a commercial company (Phalanx Biotech Group, Hsinchu, Taiwan) using Human v7.1 miRNA OneArray platform that is designed to contain 100% of miRBase 21 database.

### Luciferase reporter assay

The assay was performed as previously described.^17, 18, 19^

### Bisulfite sequencing analysis

The methylation status of miR-454-3p promoter was determined by BSP. miR-454-3p DNA was extracted using a DNA kit (Qiagen 51306, Duesseldorf, Germany), and 2 *μ*g of DNA was subjected to bisulfite conversion using an EpiTect Bisulfite Kit (59104, Qiagen, Germany) according to the manufacturer's instructions. The transformed DNA was then PCR-amplified using the TaKaRa rTaq Kit (R001B, TaKaRa, Dalian, China). The PCR amplification products were sequenced by Invitrogen Corporation, Shanghai, China.

### Generation of xenografts

Six-week-old BALB/c female athymic nude mice (Vitalriver, Beijing, China) were subcutaneously injected in the right flank with cells (2 × 10^6^ in 0.1 ml PBS). The volume of xenografts was measured every 5 days (tumor volume=(length × width^2^)/2). The mice were killed after 30 days. Tumor samples were processed for routine IHC.

### Statistical analysis

Data are expressed as the mean±S.E.M. of at least three independent experiments, and statistical evaluation was performed using one-way analysis of variance (ANOVA) or Student′s *t*-tests. Values of *P*<0.05 were considered statistically significant.

## Figures and Tables

**Table 1:** The relationship between HOTAIR expression and clinicopathological variables of chondrosarcoma

**Items**	**HOTAIR**		***P*****-value**
	**Low**	**High**	
All cases	12	12	
*Age*			0.321
>18	8	6	
<18	4	6	
			
*Gender*			0.252
Male	9	8	
Female	3	4	
			
*Anatomical location*			0.182
Limb bone	5	4	
Axial bone	7	8	
			
*Grade of tumor*			0.012
Low (grade I)	10	3	
High (grades II+III)	2	9	
