# Supplementary material for: Knockdown of long non-coding RNA HOTAIR increases miR-454-3p by targeting Stat3 and Atg12 to inhibit chondrosarcoma growth
Source: Cell Death Dis. 2017 Feb 9;8(2):e2605–. doi: 10.1038/cddis.2017.31 (PMC5386479; doi:10.1038/cddis.2017.31)
Supplement: Supplementary Table S1 [file cddis201731x1.docx]

**Table S1**

The sequences targeting HOTAIR and EZH2.

| siHOTAIR#1 | 5′--GAACGGGAGUACAGAGAGAUU-3′ |
| --- | --- |
| siHOTAIR#2 | 5′- GAACGGGAGUACAGAGAGA-3′ |
| siHOTAIR#3 | 5′- CCACAUGAACGCCCAGAGA-3′ |
| siEZH2#1 | 5′- GAAUGGAAACAGCGAAGGATT -3′ |
| siEZH2#2 | 5′- GAAUGGAAACAGCGAAGGA -3′ |
| siEZH2#3 | 5′- GUGCCCUUGTGUGAUAGCACAA -3′ |
