# Supplementary material for: Knockdown of long non-coding RNA HOTAIR increases miR-454-3p by targeting Stat3 and Atg12 to inhibit chondrosarcoma growth
Source: Cell Death Dis. 2017 Feb 9;8(2):e2605–. doi: 10.1038/cddis.2017.31 (PMC5386479; doi:10.1038/cddis.2017.31)
Supplement: Supplementary Table S2 [file cddis201731x2.docx]

**Table S2**

Primers for quantitative real-time RT-PCR

| ID | Sequence(5′-3′) |
| --- | --- |
| HOTAIR: F | GGTAGAAAAAGCAACCACGAAGC |
| HOTAIR: R | ACATAAACCTCTGTCTGTGAGTGCC |
| Stat3: F | CCCCATACCTGAAGACCAAG |
| Stat3: R | GGACTCAAACTGCCCTCCT |
| Atg12: F | AGGTCTGTAGTCGCGGAGAA |
| Atg12: R | AGCTTTCCCTTAGCAGTCTTCA |
| GAPDH: F | ACACCCACTCCTCCACCTTT |
| GAPDH: R | TTACTCCTTGGAGGCCATGT |
| U6: F | CTCGCTTCGGCAGCACA |
| U6: R | AACGCTTCACGAATTTGCGT |
